# Daily and seasonal variabilities of thermal stress (based on the UTCI) in air masses typical for Central Europe: an example from Warsaw

**DOI:** 10.1007/s00484-020-01997-8

**Published:** 2020-09-07

**Authors:** Monika Okoniewska

**Affiliations:** Institute of Geography, Kazimierz Wielki University, Koscielecki Sq. 8, 85-033 Bydgoszcz, Poland

**Keywords:** Thermal stress, UTCI, Daily and seasonal changes, Air masses, Central Europe

## Abstract

The objective of research involved the comparison of daily and seasonal courses of thermal stress occurring in Central Europe depending on the inflowing air mass. The analysis used data from Warsaw (1991–2000), including air temperature (°C), water vapour pressure (hPa), wind speed (m s^−1^) and cloud cover (%). Universal thermal climate index was calculated and subsequently averaged for the individual months and four types of atmospheric air masses: polar maritime (mP), arctic(A), polar continental (cP) and tropical (T). The studies analysed differences in daily patterns of the averaged values of universal thermal climate index between air masses and determined the frequency of days with various types of thermal stress in individual air masses. The analyses indicated that under the conditions of Central Europe, the highest daily variance of biothermal conditions occurs between the masses of cP and T in the spring and autumn. Considerably greater diversity of biothermal conditions was observed between the masses during daytime compared with nighttime, especially in the warm half of the year. The thermal stress, which can be encountered in Central Europe, ranges from an “extreme cold stress” in winter at night and early morning hours to “very strong heat stress” in summer at noon. Extreme thermal stress is related primarily to the masses of cP, A and T. The most optimal biothermal conditions occur during the advection of mP air.

**Electronic supplementary material:**

The online version of this article (10.1007/s00484-020-01997-8) contains supplementary material, which is available to authorized users.

## Introduction

The area of Central Europe is located in zone influenced by air masses from the west—flowing from over the Atlantic Ocean, as well as from the east, from the Asian continent. Moreover, air masses from the north and the south collide above it. Such diverse advection of different masses of atmospheric air is associated with the considerable diversity of climatic (Bartoszek 2017; Bąkowska 2005; Kożuchowski and Żmudzka 2002; Nowosad and Stach 2014; Sepp and Jaagus 2002; Ustrnul 2006; Ustrnul and Czekierda 2002; Ustrnul and Wypych 2011) and bioclimatic conditions (Bąkowska and Więcław 2009; Błażejczyk et al. 2003; Nowosad et al. 2013; Okoniewska 2016).

Because each of the air masses are characterised by different physical conditions, shaping other weather situations at the individual times of the year and day, one should also expect considerable variability of human body thermal stress during the advection of various air masses, which will manifest itself in both seasonal and diurnal cycles. Therefore, a comparison of bioclimatic characteristics of various times of the year and day in each of the masses inflowing over the area of Central Europe will allow determining which type of atmospheric air inflowing at given time is related to particularly burdensome biothermal conditions, and which masses exert a smaller stress. Therefore, the objective of this research involved the comparison of daily and yearly course of human body thermal stress occurring in Central Europe depending on the inflowing air mass.

Universal thermal climate index was used in the analysis due to the fact that it is an index objectively assessing the existing stress affecting human body by heat or cold, regardless of subjective individual or populational features. In modern bioclimatic studies, universal thermal climate index (UTCI) is used in the temporal and spatial analysis of biothermal conditions of various areas, ranging from the local scale (including urbanized areas), as well as covering the entire country or geographical region. Among the works covering a smaller spatial scope, there are research on the Polish coast zone conducted by Chabior (2011), Nidzgórska-Lencewicz and Mąkosza (2013), Nidzgórska-Lencewicz (2015), Półrolniczak et al. (2016), Kolendowicz et al. (2017) or Koźmiński and Michalska (2019). In turn, Błażejczyk et al. (2014) as well as Lindner (2011) based on the UTCI characterized the thermal stress in Warsaw, Mąkosza’s research (2013) focused on the analysis of biometeorological conditions of the Lubuskie Voivodeship, while the analyses of Dobek et al. (2013) discussed the issue of spatial variability of thermal stress during various weather situations in Lublin. Works on biothermal conditions prevailing throughout the country or even crossing the borders of one country include, among others, the researches of Błażejczyk et al. (2015) assessing the variability of biometeorological conditions in the region of Central and Southern Europe or the study of Błażejczyk and Błażejczyk (2014) for selected European cities. It should also be mentioned that Nemeth (2011) research related to changes in biothermal conditions in Hungary or Bleta et al. (2014) regarding Crete. Quansheng et al. (2016) investigated the spatial occurrence of biothermal conditions in China, and Brӧde et al. (2011) taked the problem of forecasting thermal comfort on the example of southern Brazil. In turn, studies by Okoniewska and Więcław (2013) referred to the variability of biothermal conditions in Poland in noon hours.

The research also deals with the issue of the relationship between heat stress and atmospheric circulation. In this trend, Katavoutas and Flocas (2018) analysed metropolitan city of Athens in Greece, Rozbicka and Rozbicki (2018) in the south area of Warsaw and Gargol and Jakubowska (2014) in Cracow. Nowosad et al. (2013) as well as Bartoszek et al. (2017) conducted similar studies for Lesko and Lublin, while Bryś and Ojrzyńska (2016) for Wrocław. The analysis of the impact of individual air masses on the formation of thermal stress was initiated by the author in earlier studies. These works focused on the analysis of diurnal variability of biothermal conditions (using also other indices as STI, PhS, Iclp, MHR, HSI, W_Sult, PST) in individual cities like Bydgoszcz (Bąkowska and Więcław 2009; Więcław and Okoniewska 2015) or Mrocza (Okoniewska 2015) and in several cities representing different regions of Poland (Bąkowska 2010; Okoniewska 2013). The relationship between atmospheric circulation and diurnal variability of biothermal conditions was discussed by Okoniewska (2016), who made detailed analysis of the diurnal course of indices (PST, UTCI, MHR) in various air masses in selected Polish cities.

The presented description develops, initiated in earlier works, research on the diurnal characteristic of biothermal conditions depending on the inflow of atmospheric air masses. Compared with previous studies, however, it focuses not so much on the index values themselves in different air masses, but on showing the amount of biothermal conditions differentiation, which occur depending on the type of advection. The inclusion of all months in the study allows obtaining information on the degree of thermal stress differentiation in both diurnal and seasonal distribution, while the analysis of both averages as well as maximum and minimum values of the UTCI allows research of variability in both average and extreme biothermal conditions.

**Fig. 1:**
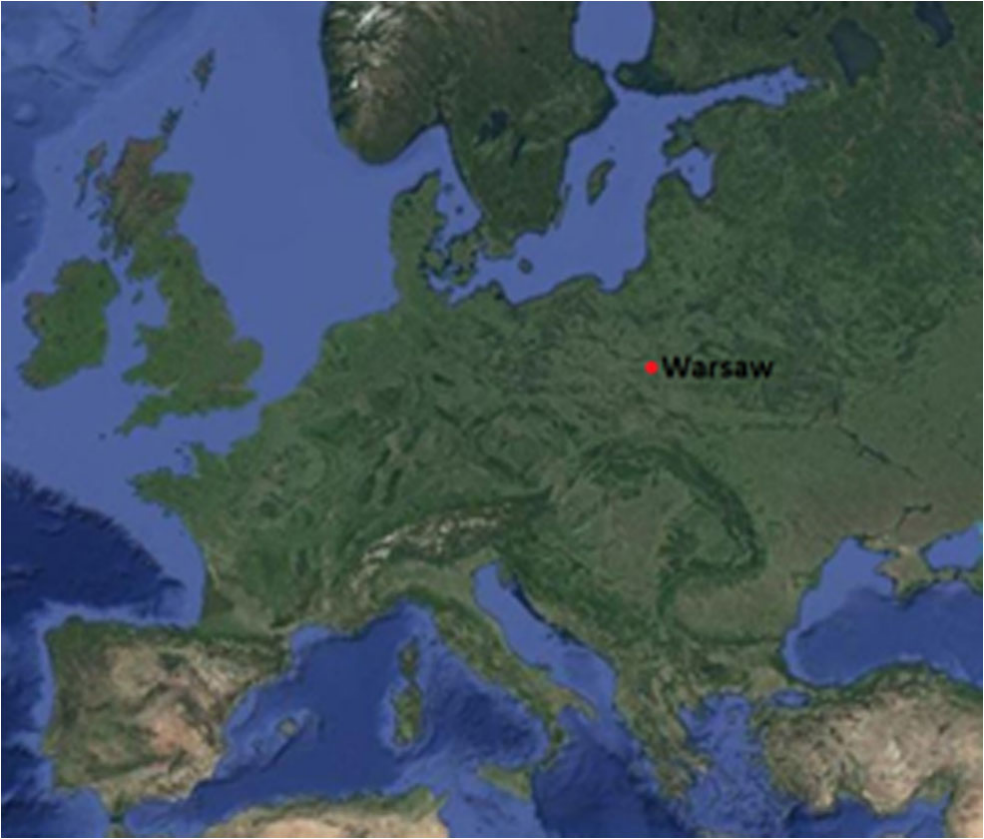
Location of the study area (www.google.pl/maps)

For current analysis, there was selected Warsaw located in the Central Mazovian Lowland, which are a subprovince of the North European Plain (Kondracki 2002) (Fig. 1). The meteorological station in Warsaw (Warsaw-Okecie—WMO 12375) is located on the tarmac of the Okęcie Airport, 11 km west of the city centre (*φ*: 52° 09′, *λ*: 20° 59″, hs 106 m a.s.l).

Synoptic conditions of Warsaw are typical for Central Europe (Stopa-Boryczka et al. 2013; Więcław 2004). They are characterized by a large variability of inflowing air masses, affecting the significant differentiation of weather conditions (Bartoszek 2017; Błażejczyk 2002, 2006). In the study period (1991–2000) the most common air masses over Warsaw were polar maritime air masses, followed by arctic, then polar continental, while tropical masses flowed the least frequently (Online Resource 1). This distribution of air masses frequencies over Poland is also confirmed by studies of other authors carried out for other regions and different periods (i.e. Błażejczyk 2002, Stopa-Boryczka et.al. 2013, Szychta 2002, Więcław 2004, 2010, 2013). In the last decade of the twentieth century, compared with previous years, a decrease in the occurrence of polar maritime air was noted, while the frequency of arctic and tropical masses increased. A similar trend was shown by Błażejczyk’s (2002) studies comparing 1960s and 1990s. Also, research by Niedźwiedź (2003) conducted for southern Poland informs about a twofold increase in the frequency of arctic masses in the second half of the twentieth century and an increase in the incidence of tropical air in the 1990s. Preliminary studies on the UTCI (for the noon hours) showed that in the 1991–2000 in Warsaw, the average UTCI were very close to the long-term average from 1961 to 2019. Comparing the last decade of the twentieth century with the other decades of 1961–2019 period, it can be stated that in the 1990s UTCI values were higher than in previous decades, which is also confirmed by the author’s previous research (Okoniewska and Więcław 2013), but lower than in the years 2000–2019. This trend was also confirmed by the analysis of the frequency of thermal stress (Online Resource 1).

## Material and methods

In the analysis data from station Warsaw-Okecie (of the Polish Institute of Meteorology and Water Management—National Research Institute—IMGW-PIB) from the years 1991–2000 (https//dane.imgw.pl/data/dane_pomiarowo_obserwacyjne/), including the following meteorological parameters: air temperature (°C), water vapour pressure (hPa), wind speed (ms^−1^) and cloud cover (%), were used. The data originated from eight observation times: 0 a.m., 3 a.m., 6 a.m., 9 a.m., 12 p.m., 3 p.m., 6 p.m., and 9 p.m. UTC. Based on them, using the BioKlima ver. 2.6 software (http://www.igipz.pan.pl/bioklima.html), the universal thermal climate index was calculated and subsequently averaged for the individual months and four types of atmospheric air masses.

The universal thermal climate index (*UTCI*—in degree Celsius) is defined as equivalent air temperature at which under reference conditions the basic physiological parameters of the body take on the same values as under actual conditions. It is based on an analysis of human thermal balance, performed using a multi-node heat exchange model—the Fiala model (Błażejczyk et al. 2010; Brӧde et al. 2012; Błażejczyk et al. 2013; Fiala et al. 2012). It measures objective changes in physiological parameters of the body, occurring due to the impact of the conditions of atmospheric environment. The thermal stress categories are presented in the supplementary material (Online Resource 2).

Information related to the inflow of atmospheric air masses above the area of Central Europe originated from an analysis of surface synoptic maps of Europe for 12 a.m. UTC, published in the Daily Meteorological Bulletin of IMGW-PIB (Daily Meteorological Bulletin (1991-2000) . Days selected for the analysis included those on which the same air mass was present all day long above the investigated area. Due to the small frequency of the occurrence of certain air masses, in the paper only four air masses have been distinguished, consisting of their individual types: arctic air (A), polar maritime air (mP), polar continental air (cP) and tropical air (T).

The paper presents an analysis of differences in diurnal courses of the averaged values of UTCI between four types of air masses in the subsequent months of the year. By using Statistica 13 (TIBCO Software Inc (2017) the surface charts were prepared to depict the discussed differences. The red colour indicates the advantage of the UTCI value in the first air mass listed on the graph; the blue shows the dominance of the index in the second mass. When determining differences in the values of UTCI between air masses, the assumed criterion involved the frequency of occurrence of given mass; i.e. UTCI value of the mass occurring less frequently was subtracted from the index value of the mass occurring more frequently.

Moreover, differences in averaged extreme values of the index were calculated in air masses. The average extreme values were determined based on adjustment to data from eight time points of an asymmetrical function with a form like in Eq. 1.

1 $$ y=\mathrm{asin}\left( bx-c\right)\exp \frac{\mathrm{d}{\left(x-e\right)}^2}{f}+g $$

wherein *y* is the given variable (UTCI) as a function of time *x*, *a*–*g* are parameters of adjustment of measurement data to the above function.

It is an asymmetrical function similar in form to the theoretical model of heat transport, used to predict the daily course of soil temperature (Lei et al. 2010). In accordance with the research carried out by Rojek and Rojek (2004) modelling is a good method to reconstruct the daily course of meteorological parameters with knowledge of values from several measurement terms.

In order to relate calculated differences in the index to actual responses of human thermoregulatory system to the occurring biothermal conditions, the frequency of days with various types of thermal stress was determined in individual air masses. Due to the fact that apart from days with the presence of the same air mass for the whole day, there were also days with atmospheric fronts, not categorised in the present analyses; the graphs present the percentage of days with given thermal stress in four air masses and among days with undefined air masses.

## Results

### Differences in the daily course of UTCI in various air masses

#### mP-A

**Fig. 2:**
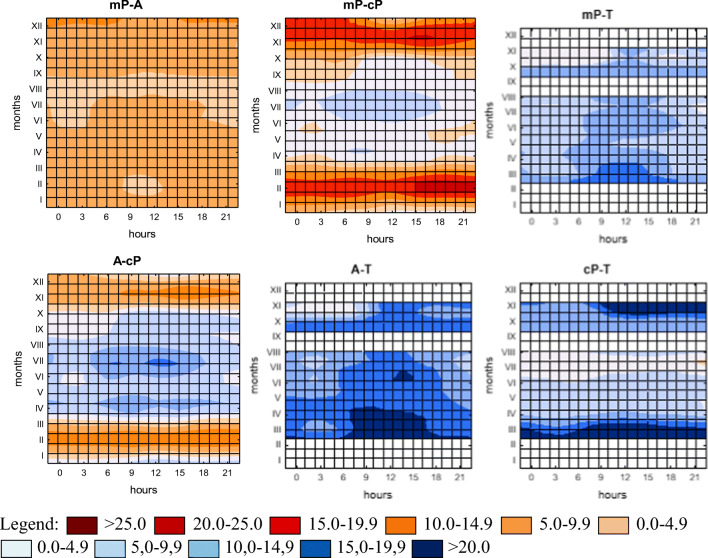
Differences (°C) between averaged values of UTCI in Warsaw in individual air masses in the consecutive months of year (1991–2000)

Biothermal contrasts between polar maritime and arctic air masses, which are the most frequent in Central Europe, are the lowest of all studied differences. MP masses are warmer over the whole year, with their smallest prevalence being recorded in August. Virtually all year long, the advection of polar maritime air makes the UTCI higher by about 5.0–7.0 °C compared with A masses. The highest variation occurs in December, when deviation in the mP mass ranges from 7.0 to 10.0 °C, while in August it is only 3.0 °C (Fig. 2). Almost for the whole year, there is also a characteristic tendency for higher biothermal diversity of both masses at nighttime, and a lower one during the day.

#### mP-cP

Masses of polar maritime air are warmer than cP masses in a period from October to March, while in October between 9 a.m. and 3 p.m. the UTCI is still slightly (by 1.0 to 3.0 °C) higher in cP air. Differences between both masses in the cold half of the year range between about 1.0 °C and even 22.0 °C, being highest in February, November and December, especially at nighttime. February is a month in which cP masses are particularly cold compared with mP air, when for the whole day UTCI is lower by 17.0 °C, and between 3 p.m. and 9 p.m., as well as at 6 a.m., even by 21.0 °C. In the warm half-year, higher values of UTCI are visible during the advection of polar continental masses, especially at daytime. The greatest differences between both masses occur in July between 6 a.m. and 6 p.m., when they exceed 5.0 °C, reaching their maximum of almost 10.0 °C at noon. May features the lowest daily variation, when for the whole day differences do not exceed 2.0 °C, and from 12 p.m. to 9 p.m. they do not increase above 1.0 °C (Fig. 2).

#### mP-T

March sees the highest diversity of biothermal conditions between T and mP air. Between 9 a.m. and 3 p.m., differences in the values of UTCI slightly exceed 15.0 °C. From April to August, values at the same hours range from about 8 to almost 14.0 °C, with higher values at daytime and lower at night. In late autumn (in November), between midnight and 6 a.m. the values of the index in both masses become almost equal; however, they indicate minor prevalence in mP masses, which seems to be caused by the presence of a thicker cloud cover in the Atlantic mass, limiting free radiation of heat. At noon in this period, the prevalence of T masses is clearly pronounced; UTCI can be higher even by 13.0 °C (Fig. 2).

#### A-cP

Almost in the whole period from November to March, higher values of UTCI (from approximately 5.0 to slightly over 10.0 °C) are recorded in arctic masses rather than in cP. The greatest difference is observed in February, when during the day UTCI in cP masses are lower by over 12.0 °C, and about 9 a.m. even by 14.6 °C. From April to October, UTCI is definitely higher in cP air. In the spring, this prevalence is considerably more pronounced compared with autumn and it ranges from about 9.0 °C at nighttime to almost 14.0 °C at 9 a.m. (April), while autumn is characterised by the prevalence of cP masses amounting to less than 2.0 °C at night and only 7.0–8.0 °C at hours around noon (October). The highest variation during the year is recorded in July, when between 6 a.m. and 3 p.m. in cP air UTCI is higher by an average of about 15.0 °C, and by almost 10 °C at night (Fig. 2).

#### A-T

The prevalence of tropical air masses over arctic air is more pronounced during daytime. In March and April, differences in UTCI between 9 a.m. and 3 p.m. exceed 20.0 °C, with highest increase in March (slightly above 23.0 °C). Moreover, in June, October and November at noon they approach 20.0 °C. At night, the greatest differences in the UTCI in the air masses in question occur in April, when between 9 p.m. and 6 a.m. they exceed 16.0 °C, and at 6 a.m. they reach almost 20.0 °C. The smallest contrasts occur in November between midnight and 6 a.m. (Fig. 2).

#### cP-T

The greatest differences between tropical and polar continental air occur in March and in November, in particular between 9 a.m. and 9 p.m., when the average value of UTCI in the T mass is higher than 20.0 °C (except 9 a.m. in November). The greatest contrast occurs in March at 9 a.m., with UTCI difference slightly exceeding 27.0 °C, and in November between 12 p.m. and 3 p.m. - above 25.0 °C. In summer, the differences between these masses are obscured (do not exceed 10.0 °C), nonetheless indicating slightly warmer conditions in tropical air (Fig. 2).

### Differences in extreme values of UTCI in various air masses

The winter period is characterised by higher variation of daily minimum UTCI values between the investigated air masses comparing to the remaining seasons. The greatest deviation occurs in February, when the index in mP air is higher by 20.5 °C than the value recorded in cP mass (Table 1). This leads to change in from “moderate” to “very strong cold stress.” Differences are also great between mP and cP masses near the end of the year, when they mean a change in the stress affecting human body from “moderate” to “strong cold stress.” When comparing two cool air masses in winter and autumn (A and cP), it can be concluded that daily minima from November to March are definitely lower in the cP mass. Particularly great differences are recorded in February, when the prevalence of daily minimum values in continental air is close to 14.0 °C (Table 1) and it means a difference in the perception of cold stress from a very high (in cP mass) to a high level (in A mass).

**Table 1:** Averaged daily minima and maxima in various air masses and differences in the values of daily minima and maxima of UTCI between masses in the consecutive months in Warsaw (1991–2000)

Month	1	2	3	4	5	6	7	8	9	10	11	12
Minima												
mP	− 11.8	− 11.7	− 8.5	− 2.4	− 0.2	6.5	9.1	9.2	5.4	− 2.7	− 6.5	− 12.9
A	− 18.8	− 18.4	− 13.3	− 10.3	− 2.1	3.5	5.4	5.5	− 0.3	− 8.3	− 13.3	− 19.9
cP	− 21.3	− 32.2	− 17.1	− 1.2	5.4	7.3	15.0	12.6	2.9	− 7.5	− 19.9	− 26.8
T	-	-	0.4	6.8	12.7	14.1	16.2	15.9	-	5.8	− 7.3	
mP-A	7.0A	6.7A	4.8A	7.9A	2.0A	3.0A	3.7A	3.7A	5.7A	5.6A	6.8A	7.0A
mP-cP	9.5cP	20.5cP	8.6cP	1.2mP	5.6mP	0.8mP	5.8mP	3.4mP	2.5cP	4.9cP	13.5cP	13.9cP
mP-T	-	-	9.0mP	9.2mP	12.8mP	7.6mP	7.1mP	6.8mP	-	8.5mP	0.8T	-
A-cP	2.5cP	13.8cP	3.8cP	9.1A	7.5A	3.9A	9.5A	7.1A	3.2A	0.8A	6.6cP	6.9cP
A-T	-	-	13.8A	17.1A	14.8A	10.6A	10.8A	10.5A	-	14.1A	6.0A	-
cP-T	-	-	17.6cP	8.0cP	7.3cP	6.8cP	1.2cP	3.3cP	-	13.4cP	12.7cP	-
Maxima												
mP	− 8.5	− 4.6	− 1.7	9.2	13.9	15.6	20.1	18.9	15.1	5.8	− 2.0	− 9.7
A	− 13.7	− 9.2	− 4.5	3.0	12.6	12.8	16.0	17.9	10.8	1.7	− 8.8	− 14.1
cP	− 13.2	− 23.3	− 6.4	15.6	19.7	22.4	32.1	26.9	19.0	9.9	− 14.4	−19.1
T	-	-	19.4	25.5	28.0	32.5	35.3	33.5	-	20.7	10.9	-
mP-A	5.2mP	4.6mP	2.8mP	6.3mP	1.3mP	2.8mP	4.1mP	1.0mP	4.3mP	4.1mP	6.8mP	4.4mP
mP-cP	4.7mP	18.6mP	4.7mP	6.3cP	5.8cP	6.8cP	12.0cP	8.0cP	3.9cP	4.0cP	12.4mP	9.4mP
mP-T	-	-	21.1T	16.3T	14.1T	16.9T	15.2T	14.6T	-	14.9T	12.9T	-
A-cP	0.5cP	14.1A	1.9A	12.6cP	7.1cP	9.6cP	16.1cP	9.1cP	8.2cP	8.2cP	5.7A	5.0A
A-T	-	-	23.9T	22.6T	15.4T	19.6T	19.3T	15.6T	-	19.0T	19.7T	-
cP-T	-	-	25.9T	10.0T	8.3T	10.0T	3.2T	6.6T	-	10.9T	25.3T	-

Next to the value of the difference, the name of air mass is given, in which the minimum or maximum value of the index dominates

In spring, when advection of tropical air appears, the highest variance of daily minima is recorded between this mass and cP air in March (Table 1), which translates into the diversity of thermal stress from “slight cold stress” in T air to “strong cold stress” in cP mass. There is also a high diversity between T and A masses. From March to May, differences in the daily minima of UTCI exceed 13.0 °C, reaching 17.0 °C in April, which evidences the variation from “slight cold stress” in T mass to “moderate cold stress” in arctic air. A considerable diversity in minimum also appears between T and mP masses, in May translating into the variability from “moderate cold stress” for mP to “no thermal stress” in T air (Table 1).

In summer, the greatest differences (exceeding 10.0 °C) in daily minimum values of UTCI are recorded between the arctic (appearance of “slight cold stress”) and tropical air (“no thermal stress”). Major deviations are also observed between T and mP air, but they do not indicate any variation in thermal stress. Daily minima between very hot air masses in the summer (cP and T) are most diverse in June, when deviations amount to 6.8 °C, in July dropping to only 1.2 °C, shaping within a range meaning the absence of thermal stress (Table 1).

In the case of maximum daily values of UTCI, the greatest diversity between air masses occurs in transitional seasons of the year, in particular in March and November, and it refers to differences between cP and A masses and tropical air. Particularly high contrasts characterise T and cP masses, in which differences in the maximum UTCI in March are close to 26.0 °C. In November they are lower by about 0.5 °C (Table 1). However, due to the lower daily maxima of UTCI in the cP mass in November, a slightly smaller difference translates into a higher variance of UTCI, which during that time ranges from “strong cold stress” (in cP air) to “no thermal stress” (in T mass). Differences between arctic and tropical air are somewhat smaller. The maximum values of index range from 22.6 to 23.9 °C in March and April and amount to less than 20.0 °C in October and November (Table 1). They mean the variability from “moderate cold stress” to „no stress”.

In winter, due to the lack of tropical air inflow, the highest variation is observed between mP and cP air, especially in February, when the difference in UTCImax amounts to 18.6 °C (Table 1) and indicates on the variability from “moderate” to “strong cold stress.” The differences between mP and A masses are definitely smaller and range between 4.0 and 5.0 °C. For arctic and polar continental masses higher maxima are recorded in arctic air, particularly in February when they are higher by about 14.0 °C. These contrasts are blurred in January, since the daily difference in maximum amounts to 0.5 °C, indicating a slight biothermal prevalence of cP masses (Table 1).

In summer, the highest daily maximum of UTCI occurs in tropical air; therefore, the differences between this and the remaining masses are the greatest, reaching their maximum between arctic and tropical mass of up to about 19.0 °C, which translates into a change in thermal stress from “strong heat stress” to neutral conditions. There are also great differences between T and mP air, which amount to about 16.0 °C and indicate similar variability of thermal stress (Table 1).

### Frequency of days with various thermal stress affecting human body in individual air masses

The most extreme thermal stress by cold exerted on human, which can be encountered in Central Europe, involves the “extreme cold stress.” It is noted only in February and December, between 6 p.m. and 6 a.m., during the advection of cP and A air. Sometimes this type of stress appears also on days with migrating atmospheric fronts.

“Very strong cold stress” occurs primarily during cP air, usually in November, when between 6 a.m. and 6 p.m. their frequency exceeds 60%, and at noon it amounts to 100%. In winter, the frequency usually ranges between about 20 and 40%; reaching 80% only in December around noon. From December to February it is also recorded during arctic masses with a frequency between 5 and 64%, primarily in December. Occasionally “very strong cold stress” occurs also during mP air.

A “strong cold stress” is recorded relatively frequently during the migration of atmospheric fronts, when it occurs in over 50% of all cases in the cold half year. In terms of air masses, it is relatively common in arctic masses, in spring and autumn from 20 to even 60% of days. Because polar continental masses in winter are more frequently responsible for the occurrences of “very strong cold stress,” a slightly lower thermal stress with this type of airflow is noted in about 10–20% of cases in December and January, and in sporadic situations in February. On the other hand, in February much more frequently compared with other months, this type of thermal stress occurs for the whole day in the presence of mP masses, warmer at this time of the year.

Similar to “slight cold stress” the “moderate cold stress” prevails during days with a change in atmospheric masses. Both types of thermal stress occur during the whole year. The “moderate cold stress” is recorded much more frequently than “slight” during the presence of A masses, especially in transitional seasons of the year. In summer it occurs in the presence of mP masses, especially at nighttime. During the advection of air from above the Atlantic Ocean, it also occurs in winter with frequency of about 20% for the whole day, sporadically is noted in cP masses.

Polar maritime masses are usually responsible for the perception of “slight cold stress” (except for situations of atmospheric front migration), which occurs during the whole year with a frequency from about 10 to 40%. “Slight cold stress” may also occur during the advection of cool arctic air masses. This happens at hours around noon from January to April and all day long from May to October, as well as sporadically around noon in November. In spring and autumn, the inflow of cP and T masses is also responsible for the possibility of feeling “slight cold stress”.

The most advantageous biothermal conditions (“no thermal stress”) may occur in winter at hours around noon, for the whole day in the remaining part of the year, with half of their cases being related to migrating atmospheric fronts and another half to weather inside masses. From April to November, “no thermal stress” occurs during advection of mP air during the whole day, with frequency from 6 to 40%. In April, it is also noted during advection of tropical masses, and although in these masses preferable biothermal conditions may also occur in other months, in April they are observed with the highest frequency, even in 50% at midnight. In the warm half of the year, “no thermal stress” conditions also occur during the inflow of cP masses. It is recorded relatively rarely in June and July, when cP masses are responsible for conditions which exert greater stress to human. Moreover, one should notice the fact of a sporadic lack of thermal stress in the summer at hours around noon, with inflow of arctic air masses.

“Moderate heat stress” occurs between April and October at daytime. It is primarily related to inflowing tropical masses in April and October, as well as mP, cP, T masses and front weather in the remaining period. In July and August, between 9 a.m. and 3 p.m. it is more frequently recorded in polar maritime air, sometimes continental, more rarely in tropical. In turn, at 6 a.m. and 6 p.m. in August, apart from situations related to the migration of atmospheric front, it in about 30–50% of all cases occurs in T mass, usually in the evening and morning.

Between May and August “strong heat stress” is only noted from 9 a.m. to 6 p.m. They emerge during the migration of atmospheric fronts, but they are also related to tropical masses in June and August, as well as polar continental in July. Sometimes, especially in July, they are also observed during the inflow of mP masses.

“Very strong heat stress”—the most intense stress affecting body in summer, occurs between June and August at hours around noon and is related to the inflow of T masses. In July, it can also occur in situations of migrating atmospheric front (Online Resource 2).

## Conclusions and discussion

Under the climatic conditions of Central Europe, the greatest daily biothermal diversity occurs between the cP and T masses in spring and autumn. The greatest differences in minimum and maximum daily values are recorded in March. However, the longest period with considerable differences between air masses is related to tropical and arctic masses, in which deviations of UTCI from March to November (except for September) at daytime exceed 15.0 °C.

The studies indicate greater diversity of biothermal conditions between masses during daytime compared with nighttime, especially in warm half of the year. In winter, conditions perceived in the studied air masses are more similar to each other. An exception involves the variability of UTCI in mP and cP air.

The most extreme thermal stresses, which can be encountered in Central Europe, are “extreme cold stress” in winter and “very strong heat stress” in summer. Similar results for this part of European continent were produced by Kuchcik et al. (2013), Lindner (2011), and Rozbicka and Rozbicki (2018). In winter, such extreme biothermal conditions are recorded only between 6 p.m. and 6 a.m., with an inflow of frosty masses of cP or A air. This fact is also confirmed by research of Bąkowska and Więcław (2009), albeit related to subjective temperature index (STI), whose values are nonetheless the lowest at night and morning hours in polar continental and arctic air. Apart from air temperature, the authors see the reason for this in the low water vapour content, characteristic of this type of air masses, and thus in a sparse cloud cover, leading to fast radiation of heat from the ground. Strong relations between the occurrence of thermal stress and atmospheric circulation were also noted by Rozbicka and Rozbicki (2018), who considered the air inflow from the east sector to be responsible, among other things, for the occurrence of “extreme cold stress” in winter, and Więcław (2004), who concluded that high frequency of the occurrence of frosty and very frosty weather is characteristic of continental air. Similar relations between air advection and thermal stress were observed by Bąkowska (2010), who concluded the occurrence of harshest bioclimatic conditions during the inflow of cP masses. In summer, “very strong heat stress” occurs during advection of tropical air at around noon, which was also confirmed by the research of Bąkowska performed based on an analysis of the same index (2010). The research of Gargol and Jakubowska (2014) in turn links this type of thermal stress during this time with a sparse cloud cover and heavy insolation, usually occurring during an anticyclonic weather situation.

“Very strong” and “strong cold stresses” are noted in winter, spring and autumn. The first one is related mostly to the inflow of cP masses, and the second one to A masses. Both types of thermal stress can occur at any time of the day in autumn and winter. Gargol and Jakubowska (2014) have also noted in Cracow that in winter these types of thermal stresses are related to the advection of air masses from the north, as well as the south-east, additionally associating them with a high-pressure situation. According to these studies, the conditions of “very strong cold stress” are maintained no longer than for 1 day, always during situations with a high wind speed (> 5 m s^−1^) and temperature below − 5.5 °C. The research performed by Rozbicka and Rozbicki (2018) considers the anticyclonic baric situation and the inflow of air from the eastern sector to be the most responsible for the occurrence of extreme cold stress conditions .

“Moderate” and “slight cold stress” are usually associated with the situation of a passing atmospheric front. Considering weather inside the masses, “moderate cold stress” occurs primarily during the advection of arctic air in spring and autumn, as well as polar maritime air in winter. “Slight cold stress” is much more often associated with the inflow of masses from the Atlantic Ocean.

The most advantageous conditions for human are usually noted during the migration of atmospheric fronts, or during the inflow of mP masses. It is only in April when such optimal biothermal conditions occur quite frequently also in tropical air flowing from the south. The biometeorological advantageousness of mP masses is also pointed out by the research of Błażejczyk (2002) indicated that warm weather with moderate radiation stimuli, with moderate sultriness intensity but high daily thermal contrasts and physiological heat stress is related to mP air, especially its transformed type. The research of Okoniewska (2013, 2016) also claims that polar maritime masses are those characterised by the most optimal conditions of perceptible climate, not causing excessive stress by either heat or cold.

“Moderate heat stress” occurs from April to October during daytime, in these months being noted under the conditions of warm T masses. In the remaining period it occurs more often during the migration of atmospheric fronts and in mP, sometimes cP mass. The research of Więcław (2004), characterising the spatial diversity of the frequency of occurrence of weather types in individual air masses, points at mP masses as clearly responsible for the prevalence of days with moderately warm weather.

“Strong heat stress” in turn occurs only from May to August, usually between 9 a.m. and 3 p.m. It is usually related to the inflow of T air, cP air and less frequently mP. It also appears frequently during changes in the inflowing air mass. This is also confirmed by the research of Rozbicka and Rozbicki (2018), who concluded that “strong” and “very strong” heat stresses are related mainly to anticyclonic weather situations to the inflow of air from the east and south-east.

## Electronic supplementary material

ESM 1 (PDF 494 kb)

ESM 2 (PDF 407 kb)

ESM 3 (PDF 619 kb)
