# Supplementary material for: Daily and seasonal variabilities of thermal stress (based on the UTCI) in air masses typical for Central Europe: an example from Warsaw
Source: Int J Biometeorol. 2020 Sep 7;65(9):1543–52. doi: 10.1007/s00484-020-01997-8 (PMC8370898; doi:10.1007/s00484-020-01997-8)
Supplement: Supplementary file 1 — (PDF 494 kb) [file 484_2020_1997_MOESM1_ESM.pdf]

Occurrence of air masses above the area of Central Europe

Tab. 2 Number of days and frequency of occurrence for specific air masses in Central Europe (Warsaw) in the years 1991-2000.

air masses	mP		A		cP		T		undenfined air masses (passying anything front)	
month	number of days	frequency (%)	number of days	frequency (%)	number of days	frequency (%)	number of days	frequency (%)	number of days	frequency (%)
January	50	16.1	39	12.6	35	11.3	-	-	186	60.0
February	46	16.4	28	10.0	9	3.2	-	-	200	70.7
March	31	10.0	67	21.6	24	7.7	1	0.3	187	60.3
April	31	10.3	43	14.3	21	7.0	17	5.7	188	62.7
May	61	19.7	39	12.6	22	7.1	3	1.0	185	59.7
June	92	30.7	12	4.0	11	3.7	10	3.3	175	58.3
July	101	32.6	9	2.9	15	4.8	5	1.6	180	58.1
August	104	33.5	11	3.5	29	9.4	13	4.2	153	49.4
September	60	20.0	36	12.0	23	7.7	-	-	181	60.3
October	53	17.1	47	15.2	12	3.9	11	3.5	187	60.3
November	54	18.0	25	8.3	19	6.3	1	0.3	201	67.0
December	44	14.2	46	14.8	16	5.2	-	-	204	65.8
Average annual number of cases	72.7	19.9	40.2	11.0	23.6	6.5	6.1	1.7	222.7	61.0

Due to its position, the area of Central Europe is characterised by considerable variability of the inflow of atmospheric masses (Bartoszek 2017, Więcław 2004). Within the investigated period of 1991-2000, days with clearly pronounced air masses constituted 39%, while the remaining 61% of days had uncategorised air masses, when the migration of a atmospheric front was visible. The presented results indicate the prevalence of polar maritime air masses (mP), which is confirmed by the previous research of authors studying the issues of atmospheric circulation above Poland (e.g. Niedźwiedź 2003, Szychta 2002, Więcław 2004, 2010, 2013). In the studied years, these masses exhibited particularly high frequency from June to August, when slightly more than 1/3 of all days featured the advection of polar maritime air (mP). On the other hand, their lowest frequency was recorded in the spring. The second highest frequency was recorded for arctic masses (A), whose maximum value of 21.6% days occurred in March. In July, these masses were recorded with the lowest frequency – only in less than 3% of all cases. Masses of polar continental air (cP) appeared for an average of 6.5% of days in year. The frequency of their occurrence ranged between 3.2% in February and 11.3% in January. The advection of tropical masses (T) was the least frequent above Central Europe. The highest frequency of their occurrence was in April – in 5.7% cases; in January, February, September and December they did not occur at all in the investigated period (tab. 2).

Characteristics of the Universal Thermal Climate Index in the period 1991-2000

Tab. 3 Average ($UTCI_{av}$), maximum ($UTCI_{max}$) and minimum ($UTCI_{min}$) values of the UTCI in Warsaw at 12 UTC in 1961-2019 and in the following decades

Years	$UTCI_{av}$	$UTCI_{max}$	$UTCI_{min}$
1961-1970	3,29	37,81	-38,17
1971-1980	3,58	36,63	-51,90
1981-1990	4,28	36,36	-42,34
1991-2000	4,99	41,10	-38,24
2001-2010	6,00	37,98	-40,78
2011-2019	7,92	40,44	-44,65
1961-2019	4,96	41,10	-51,90

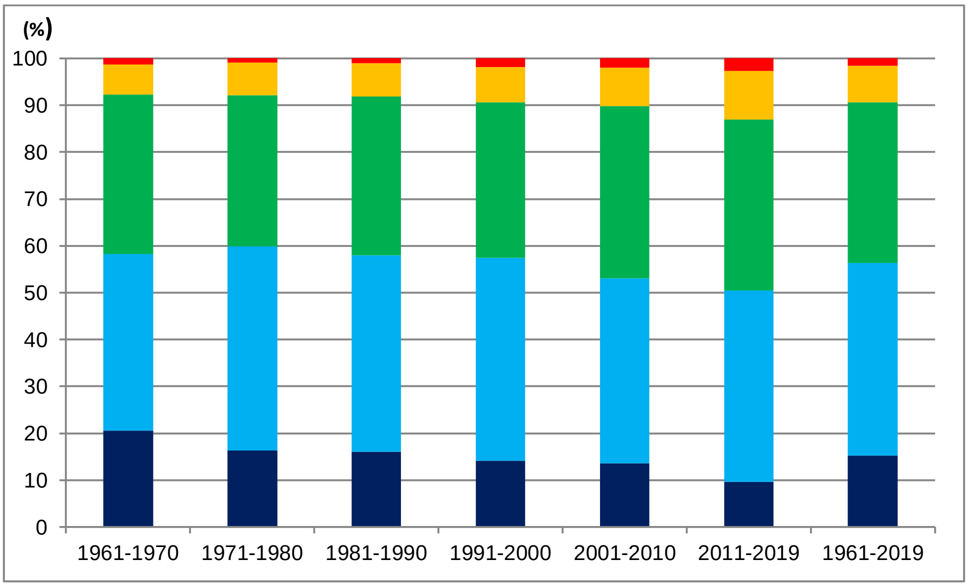

Legend: extreme cold stress strong and very strong cold stress moderate and slight cold stress no thermal stress moderate heat stress strong and very strong heat stress

Fig. 3 Frequency of occurrence of thermal stress based on UTCI in Warsaw at 12 UTC in 1961-2019 and in the following decades

The average values of the UTCI in the years 1991-2000 in the noon hours were very close to the long-term average from the period 1961-2019, amounting to nearly 5.0 °C. The analyzes also showed that in the last decade of the 20th century the average values were higher than in previous years, but definitely lower than in the period 2011-2019, when the average was 7.92 °C. In addition, the highest maximum value (41.1 °C) from 1961-2019 was recorded in 1991-2000. In the same decade, at noon, the second highest index in the entire period under review was recorded, which was -38.24 °C and was lower than the highest by less than 0.1 °C (tab. 3).

Also, the frequency of the occurrence of thermal stress on the human body at 12 UTC in the 1990s was very similar to the average incidence throughout the 1961-2019 period. Most often at this

time, „moderate” and „light cold stress” was recorded, which in 1991-2000 occurred in 43.3% of cases, while in the whole period it occurred with a frequency of 41.1%. No thermal stress in the last decade of the 20th century, as well as in 1961-2019 took place in just over 1/3 of the situation. The frequency of the appearance of more extreme thermal stress, such as „strong” and „very strong cold” or „heat stress”, also in 1991-2000 was very similar to the turnout for the entire 1961-2019 years and amounted to 14.2 and 1.9%, respectively. Comparing the results of the occurrence of thermal stress at noon in the 90s to the other decades of the 1961-2019 period, it can be concluded that the end of the 20th century was characterized by a slightly higher incidence of „moderate”, „strong” and „very strong heat stress” than previous years and at the same time lower than the the first two decades of the 21st century (fig. 3).
