# Supplementary material for: Daily and seasonal variabilities of thermal stress (based on the UTCI) in air masses typical for Central Europe: an example from Warsaw
Source: Int J Biometeorol. 2020 Sep 7;65(9):1543–52. doi: 10.1007/s00484-020-01997-8 (PMC8370898; doi:10.1007/s00484-020-01997-8)
Supplement: Supplementary file 2 — (PDF 407 kb) [file 484_2020_1997_MOESM2_ESM.pdf]

Tab. 3 Thermal stress categories based on Universal Thermal Climate Index (Błażejczyk et. al. 2010)

UTCI (°C) range	Stress category
> 46.0	extreme heat stress
38.1 to 46.0	very strong heat stress
32.1 to 38.0	strong heat stress
26.1 to 32.0	moderate heat stress
9.1 to 26.0	no thermal stress
0.1 to 9.0	slight cold stress
-13.0 to 0.0	moderate cold stress
-27.0 to -12.9	strong cold stress
-40.0 to -26.9	very strong cold stress
< -40.0	extreme cold stress
