# Supplementary material for: Daily and seasonal variabilities of thermal stress (based on the UTCI) in air masses typical for Central Europe: an example from Warsaw
Source: Int J Biometeorol. 2020 Sep 7;65(9):1543–52. doi: 10.1007/s00484-020-01997-8 (PMC8370898; doi:10.1007/s00484-020-01997-8)
Supplement: Supplementary file 3 — (PDF 619 kb) [file 484_2020_1997_MOESM3_ESM.pdf]

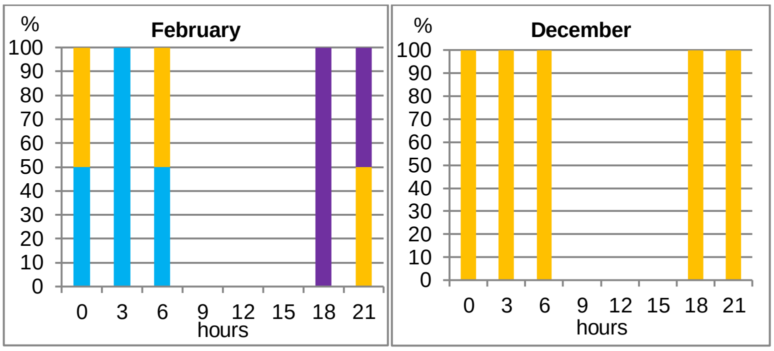

Legend: mP A cP T undefined air masses (atmospheric front passing)

Fig. 4 Frequency of days with “extreme cold stress” in Warsaw in the consecutive months of year in various air masses (1991-2000)

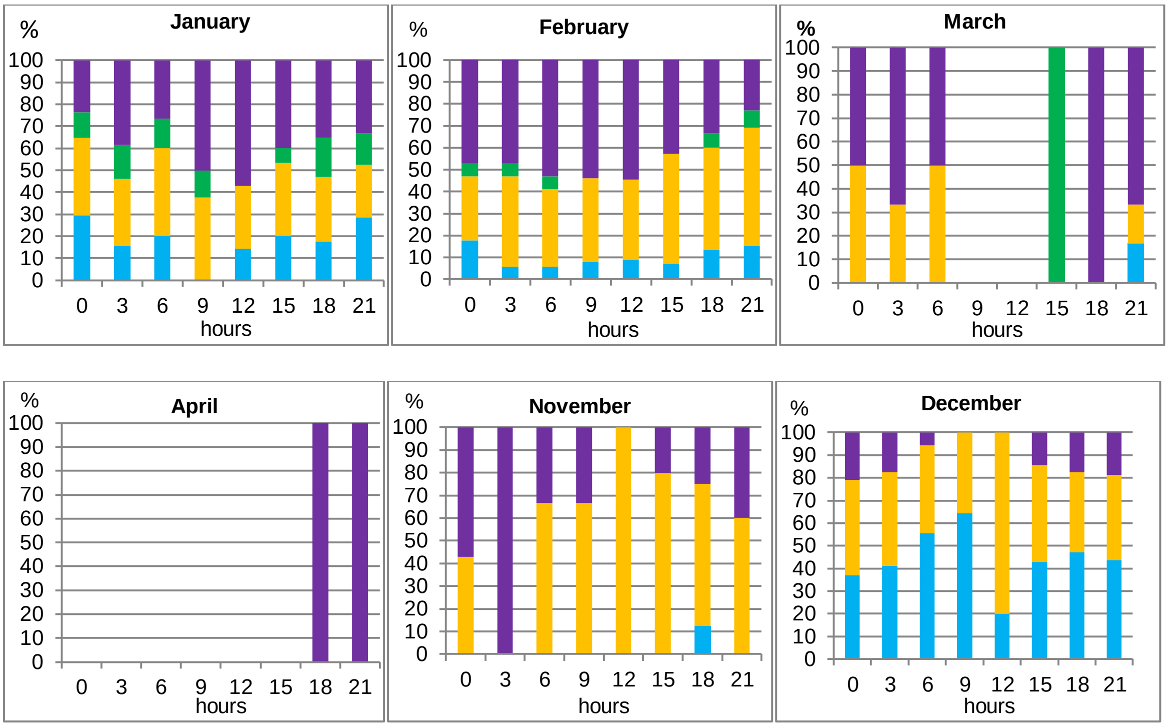

Legend as in fig. 4

Fig. 5 Frequency of days with “very strong cold stress” in Warsaw in the consecutive months of year in various air masses (1991-2000)

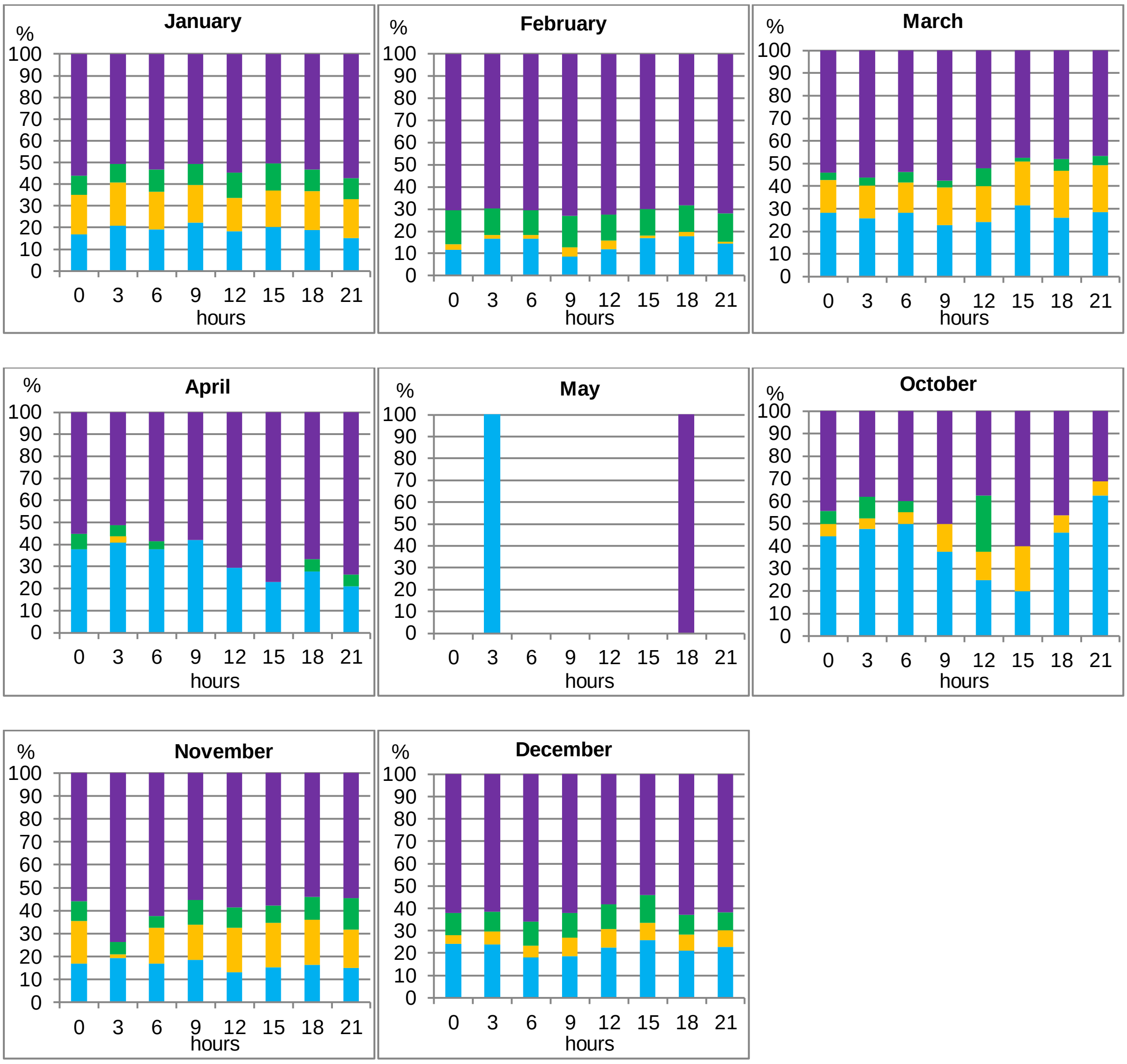

Legend as in fig. 4

Fig. 6 Frequency of days with “strong cold stress” in Warsaw in the consecutive months of year in various air masses (1991-2000)

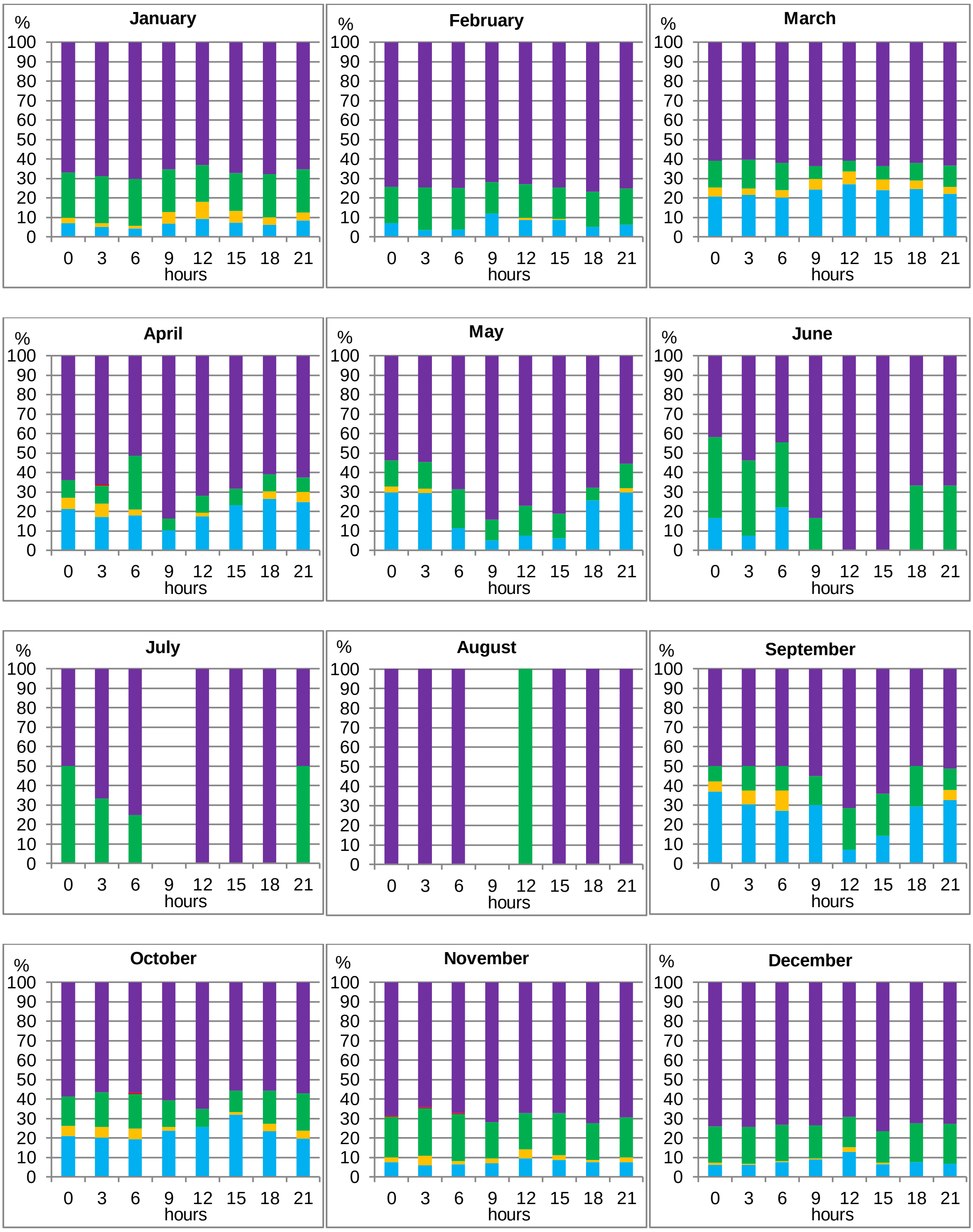

Legend as in fig. 4

Fig. 7 Frequency of days with “moderate cold stress” in Warsaw in the consecutive months of year in various air masses (1991-2000)

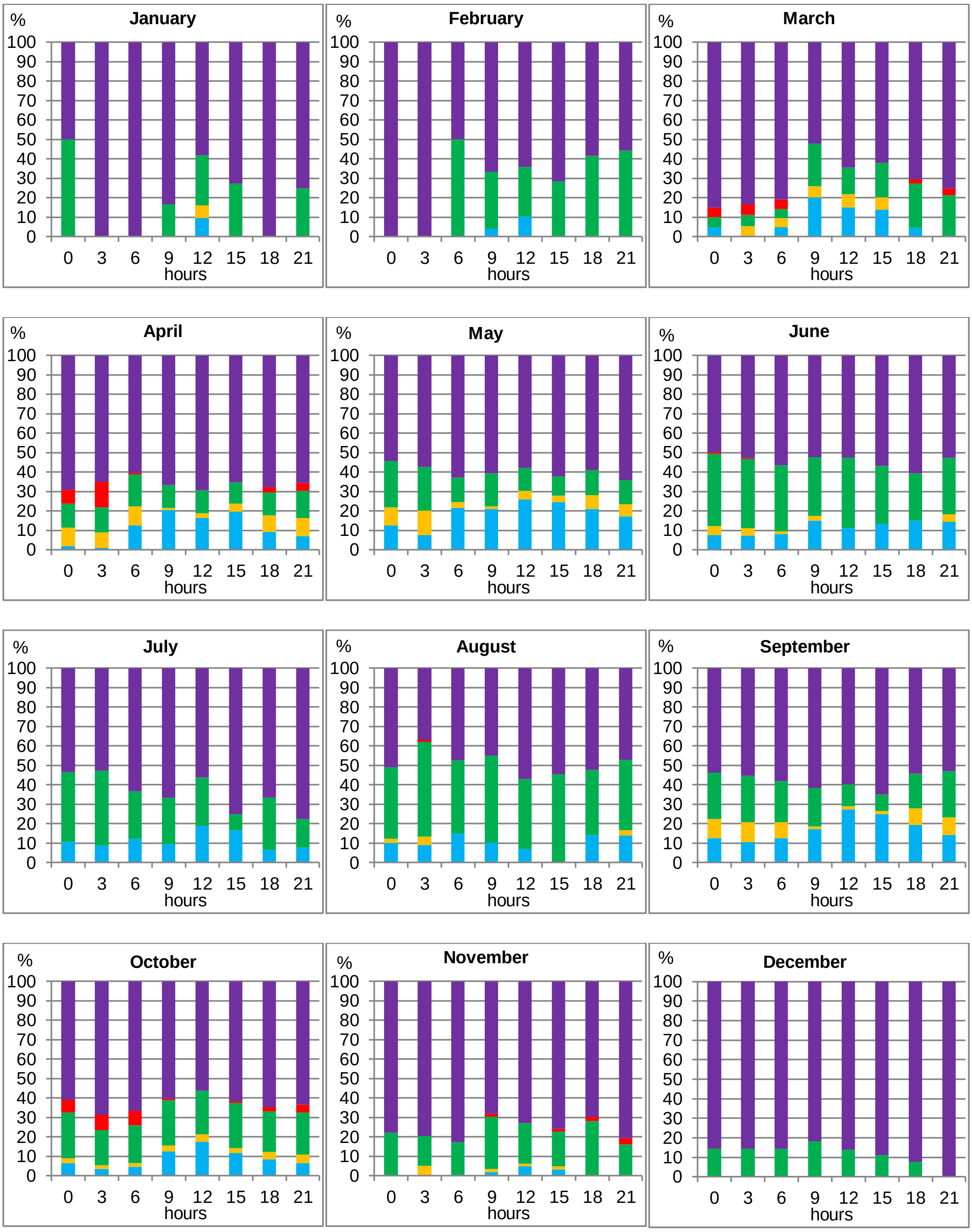

Legend as in fig. 4

Fig. 8 Frequency of days with “slight cold stress” in Warsaw in the consecutive months of year in various air masses (1991-2000)

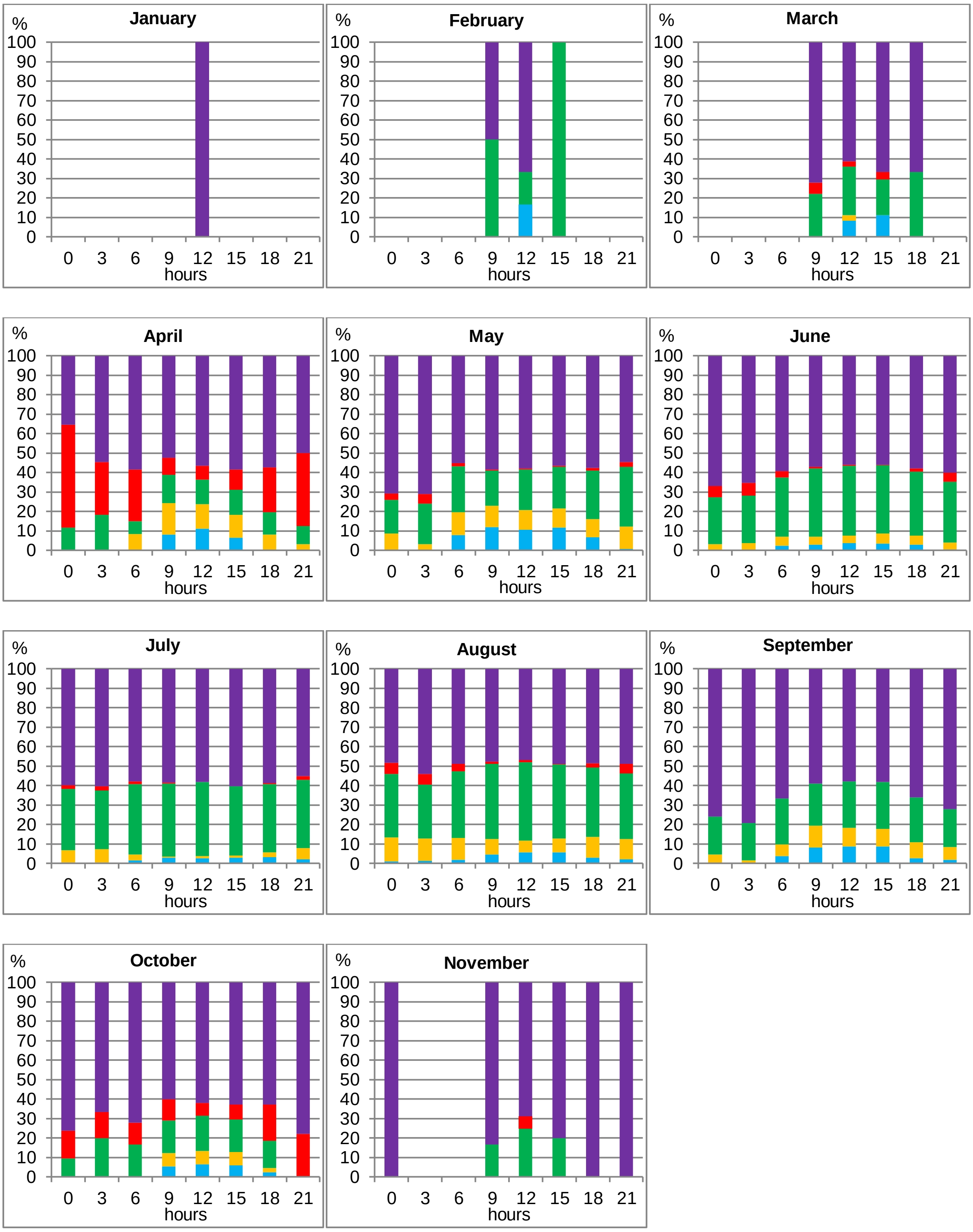

Legend as in fig. 4

Fig. 9 Frequency of days with “no thermal stress” in Warsaw in the consecutive months of year in various air masses (1991-2000)

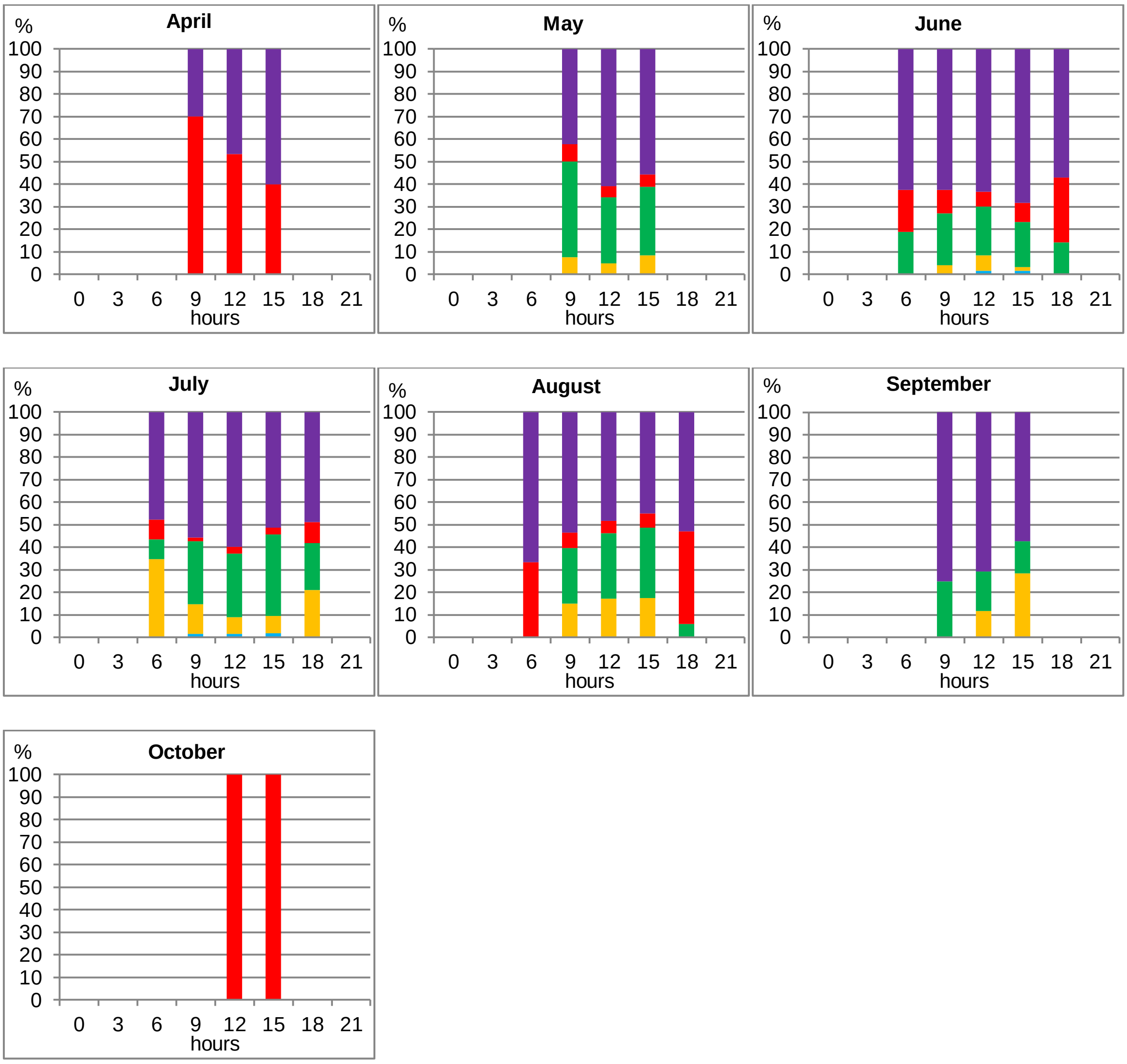

Legend as in fig. 4

Fig. 10 Frequency of days with “moderate heat stress” in Warsaw in the consecutive months of year in various air masses (1991-2000)

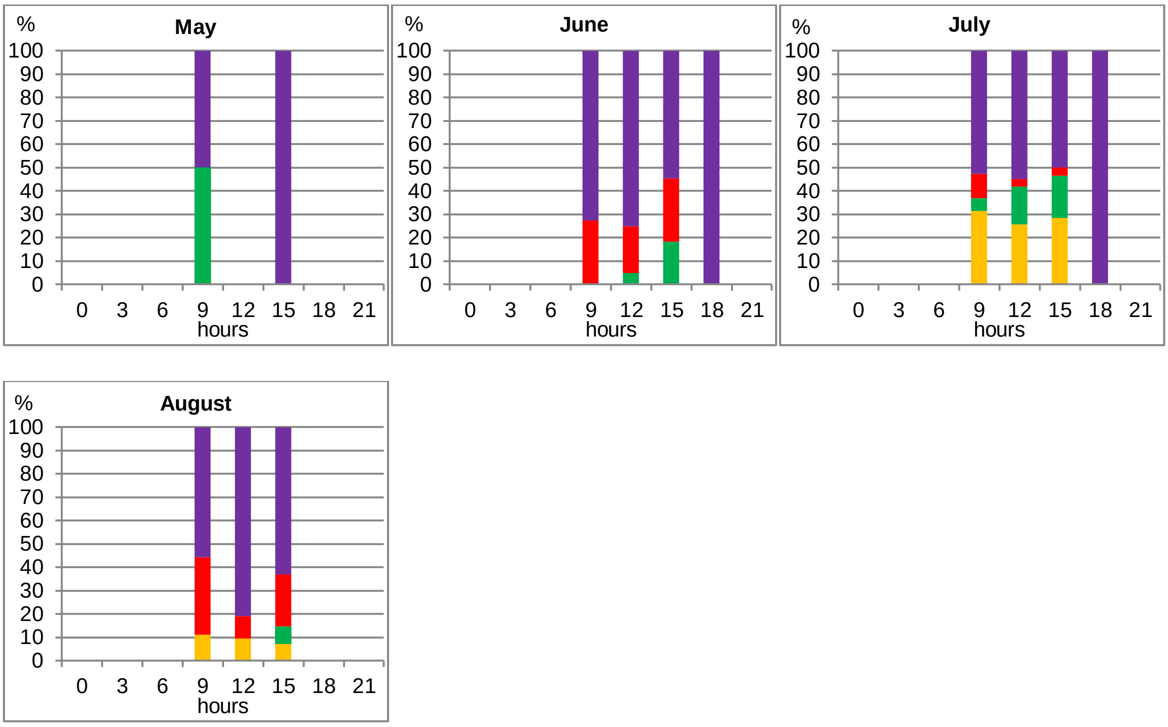

Legend as in fig. 4

Fig. 11 Frequency of days with “strong heat stress” in Warsaw in the consecutive months of year in various air masses (1991-2000)

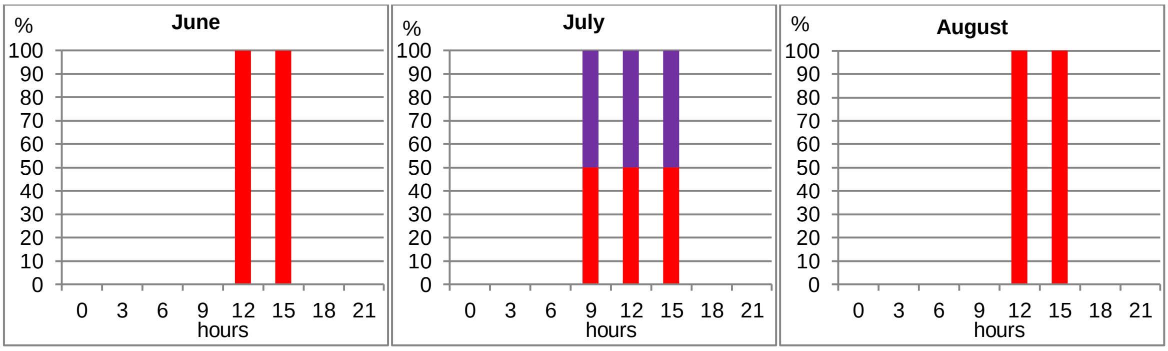

Legend as in fig. 4

Fig. 12 Frequency of days with “very strong heat stress” in Warsaw in the consecutive months of year in various air masses (1991-2000)
